# Risk Factors for African Tick-Bite Fever in Rural Central Africa

**DOI:** 10.4269/ajtmh.2011.10-0191

**Published:** 2011-04-05

**Authors:** Lucy M. Ndip, Hope H. Biswas, Landry E. Nfonsam, Matthew LeBreton, Roland N. Ndip, Marie A. Bissong, Emmanuel Mpoudi-Ngole, Cyrille Djoko, Ubald Tamoufe, A. Tassy Prosser, Donald S. Burke, Nathan D. Wolfe

**Affiliations:** Department of Biochemistry and Microbiology, University of Buea, Buea, Cameroon; Department of Epidemiology, and Department of International Health, Bloomberg School of Public Health, Johns Hopkins University, Baltimore, Maryland; Global Viral Forecasting Initiative, San Francisco, California; Johns Hopkins Cameroon Program/CRESAR, BP 7039 Yaoundé, Cameroon; Department of Biochemistry and Microbiology, University of Fort Hare, Alice, South Africa; Department of Epidemiology, University of Pittsburgh Graduate School of Public Health, Pittsburgh, Pennsylvania; Program in Human Biology, Stanford University, Stanford, California

## Abstract

African tick-bite fever is an emerging infectious disease caused by the spotted fever group *Rickettsia*, *Rickettsia africae,* and is transmitted by ticks of the genus *Amblyomma.* To determine the seroprevalence of exposure to *R. africae* and risk factors associated with infection, we conducted a cross-sectional study of persons in seven rural villages in distinct ecological habitats of Cameroon. We examined 903 plasma samples by using an indirect immunofluorescence assay for antibodies to *R. africae* and analyzed demographic and occupational data collected from questionnaires. Of the 903 persons tested, 243 (26.9%) had IgG/IgM/IgA reactive with *R. africae*. Persons from four of the seven village sites were significantly more likely to be seropositive (*P* < 0.05), and lowland forest sites tended to have higher seroprevalences. These results suggest that African tick-bite fever is common in adults in rural areas of Cameroon and that ecological factors may play a role in the acquisition of *R. africae* infection.

## Introduction

African tick-bite fever (ATBF) is an emerging infectious disease endemic in sub-Saharan Africa and the most commonly encountered rickettsiosis in travel medicine.^1^ It is an acute, influenza-like syndrome that includes symptoms such as fever, nausea, headache, and myalgia,^2^ and is caused by the spotted fever group *Rickettsia*, *Rickettisia africae. Rickettsia africae* is a gram-negative obligately intracellular bacterium that is transmitted to humans through the bite of an infected tick. The first human case of ATBF was reported in 1992.^3^ Since then, *R. africae* has been detected or isolated from ticks or humans in 15 countries in Africa.^2^,^4^,^5^ In rural central Africa, *R. africae* is transmitted by *Amblyomma variegatum* ticks, which serve as vectors and reservoirs.^6^ *Rickettsia africae* infection in *Amblyomma* ticks is frequently high with a prevalence of up to 100% reported in ticks obtained in some disease-endemic countries.^7^

Studies suggest that ecological characteristics of the tick vector influence the epidemiology of this tick-borne disease.^7^–^9^ *Amblyomma variegatum* ticks occupy a wide variety of different habitats and have a preference for semi-arid and humid areas with tall grass, tree, and/or bush cover from sea level to an altitude of 2,590 meters.^10^,^11^ International travelers entering tick habitats in disease-endemic areas have been shown to be at risk of contracting ATBF,^7^,^12^ although it is unclear which habitat types may be associated with risk of ATBF. In addition to ecological characteristics of the vector, behaviors of the host may also increase risk of ATBF. In a cohort study of 940 safari travelers from Norway to South Africa, an overall attack rate of 4.0% was observed, ranging from 2% in leisure tourists to 25% in game hunters, and hunting was identified as an independent risk factor.^12^ The authors postulated that hunting involved the most extensive exposure to ground vegetation and high grass and wild ungulates and their hides. Thus, this activity was linked with the highest likelihood of encountering infected ticks.

Although a high prevalence (30–80%) of antibodies to spotted fever group rickettsiae has been shown in persons of all ages throughout Africa,^13^–^15^ little is known about the epidemiology of ATBF in indigenous populations. Nearly all acute cases of ATBF described in the medical literature have occurred in travelers from Europe or the United States.^11^,^16^,^17^ The known cases of acute ATBF diagnosed by serologic (n = 26) and molecular techniques (n = 7) in indigenous patients from Africa were only recently documented.^4^,^5^ Anecdotal reports suggest that indigenous persons may acquire infection during agricultural work through exposure to ticks on ground vegetation,^18^ but further epidemiologic research is needed to identify the risk factors associated with infection. This cross-sectional study examines the seroprevalence of antibodies to *R. africae* and risk factors for infection among healthy adults in rural villages of Cameroon.

## Materials and Methods

### Study site and population.

Seventeen rural village sites in seven of the 10 provinces in Cameroon were selected as part of a community-based human immunodeficiency virus (HIV) prevention campaign study designed to provide information and decrease transmission.^19^ The selected villages were distributed across a range of ecological habitats. Persons were healthy adults who were recruited to the study through site coordinators in rural village clinics. For inclusion in the study, persons were required to be residents of the village, greater than 15 years of age, and willing to provide a blood sample under informed consent. During the survey, a questionnaire was administered and persons were asked to provide demographic and behavioral data that included diet, travel, sexual behaviors, and occupation. After administration of the questionnaire, approximately 15 mL of whole blood was collected by venipuncture, and fresh blood was transferred to a central laboratory where plasma was separated from the blood according to standard procedures.^20^ Samples were kept frozen at ≥ –80°C until used for serologic testing.

For this rickettsial seroprevalence study, a subset of approximately 80–180 serum samples was randomly selected from persons who had undergone venipuncture at 7 of the 17 sites included in the large epidemiologic survey. Sites were selected to represent different habitats, including one lowland coastal forest site, one mangrove site, one lowland forest site, two gallery highland forest sites, one lowland equatorial forest site, and one forest-savanna mosaic site. The seven sites included one in each of the Southwest, Northwest, West, Littoral, Central, South, and East Provinces. The study protocol was reviewed and approved by the Johns Hopkins Bloomberg School of Public Health Committee for Human Research, the Cameroon National Ethical Review Board, and the HIV Tri-Services Secondary Review Board.

### Serologic testing.

*Rickettsia africae* (ESF strain) antigen slides were prepared in the Ehrlichial and Rickettsial Diseases Research Laboratory, Department of Pathology, University of Texas Medical Branch (Galveston, TX). Rickettsiae were cultured in Vero cells, and after an infection rate ≥ 70% was achieved, cells were harvested and antigen slides prepared according to standard procedures. Indirect fluorescent antibody assays were performed on the samples to detect antibodies reactive with *R. africae* as described.^17^,^21^ Serial two-fold dilutions (1:32–1:64) of human serum were prepared in phosphate-buffered saline (PBS) containing 1% bovine serum albumin (BSA) and 0.1% Tween 20. Antigen slides were blocked in PBS containing 1% BSA and 0.01% sodium azide. Ten microliters of each serum dilution were added to each well of the antigen slide and incubated in a humid chamber for 30 minutes at 37°C. Slides were subsequently washed with PBS containing 0.1% Tween 20 for 10 minutes and then washed twice in the same solution for 10 minutes. Fluorescein isothiocyanate–conjugated goat anti-human IgA, IgG, and IgM immune serum (Kirkegaard and Perry Laboratories, Gaithersburg, MD) diluted 1:100 in PBS containing 1% BSA and 0.01% Tween 20 was added to each well and incubated in a humid chamber for 30 minutes at 37°C. Slides were washed once with PBS containing 0.1% Tween 20 for 10 minutes and once with PBS containing 0.1% Tween 20 and 0.01% Evans blue for 10 minutes. The slides were blot-dried, mounted with gel mount (Biomeda Corp, Foster City, CA), and observed under a fluorescence microscope at 400× magnification. Serum samples yielding distinctly fluorescent rickettsiae at a 1:64 dilution were considered positive. Positive samples were further two-fold serially diluted 1:128–1:4,096 to determine endpoint titers.

### Statistical analysis.

Descriptive statistics were calculated for demographic, occupational, and behavioral variables. House roofing material and ceiling condition (unfinished or finished) were used to assess socioeconomic status. Occupations were categorized by their risk of contact with ticks: agricultural occupations, occupations requiring direct contact with animals, agricultural and direct contact occupations, or neither.

The seroprevalence of antibodies to *R. africae* was calculated overall and by village site, and a chi-square test was used to evaluate heterogeneity of rates among the different villages. To examine the associations between questionnaire variables and *R. africae* seropositivity, single covariate (unadjusted) logistic regression models were generated to calculate odds ratios (ORs) and 95% confidence intervals (CIs). All variables significant at *P* < 0.10 were considered for inclusion in multivariable logistic regression models. The variables were added sequentially to a model containing only *R. africae* seropositivity. Models were then evaluated using likelihood ratio tests in a forward stepwise manner to identify a parsimonious multivariable model. To adjust for potential confounding, covariates whose addition to the model changed the ORs by ≥ 10% were included in the final model. An interaction term between hunting wild game and age group was also assessed. All analyses were conducted by using Stata/SE version 10.1 software (StataCorp, College Station, TX).

## Results

### Study participants.

Demographic, occupational, and behavioral information for the 903 persons included in this study is shown in Table 1. In the aggregate results, sexes were represented approximately equally. Within the seven sites, sex was similar with the exception of three sites: Mundemba, Nyabisan, and Sobia, which had 81.3%, 10.0% and 36.3% female participants, respectively. Study participants' ages ranged from 16 to 88, years and younger age groups (16–25 and 26–35 years) were more frequently represented. Most participants had ≤ 9 years of formal education; lived in houses with corrugated tin, tile, or tar roofs with unfinished ceilings; and worked in subsistence or market agriculture. Although few participants worked in occupations involving direct contact with animals, a substantial percentage reported having hunted wild game at some time. Among the participants who reported ever having owned a wild pet, most owned a rodent or a medium-to-large herbivore.

### Seroprevalence of antibodies to *R. africae.*

Of the 903 persons, 243 (26.9%) had antibodies reactive with *R. africae* (Table 2). High seroprevalences were seen in Njikwa (51.8%), Lomie (38%), Sobia (37%), and Nyabisan (28.7%) and represented the gallery highland (Njikwa) and most of the lowland village sites (Lomie, Sobia, and Nyabisan). The lowest rate was seen in Mouanko (11.9%), a mangrove site. Seropositivity rates for each of the seven villages by village site and ecological characteristics are shown in Table 1. Seropositivity rates were significantly different among the different villages (χ^2^ = 93.3, *P* < 0.001). Most of the lowland forest sites had high seropositivity rates. However, the highest and lowest rates were found in highland gallery-forest sites. The distribution of endpoint IgG/IgM/IgA titers for *R. africae* was 64 (16.8%), 128 (27.1%), 256 (22.1%), 512 (18.1%), 1,024 (9.8%), and 2,048 (6.2%).

Unadjusted analyses of demographic, occupational, and behavioral characteristics associated with *R. africae* seropositivity showed no significant differences in seropositivity between females and males or across different age strata, with the exception of persons 36–45 and > 55 years of age, who had significantly higher odds of seropositivity (Table 3). In contrast, increasing years of education (7–9 and > 9 years) were associated with significantly lower odds of seropositivity. Lower socioeconomic status as indicated by grass or thatched house roofing material was associated with significantly higher odds of seropositivity, as was missing house roofing material.

Four of the village sites, Lomie, Nyabisan, Sobia and Njikwa, were associated with significantly higher odds of seropositivity. Participants in high-risk occupations (occupations involving only agricultural work or agricultural work and direct contact with animals) and those who reported ever having hunted wild game had significantly higher odds of seropositivity. Although owning a wild pet had no significant effect on seropositivity, those who reported having ever owned a medium-to-large herbivore, primate, or rodent had significantly increased odds of seropositivity.

In the multivariable analyses, only sex, age group, village site and ever hunted wild game were retained in the final model. There was no significant difference in seropositivity between females and males. However, participants 36–45 years of age had significantly higher odds of seropositivity; no other age group showed this effect. Participants from the villages of Lomie, Njikwa, Nyabisan, and Sobia also had significantly higher odds of seropositivity. Those persons who reported ever having hunted game had non-significantly elevated odds of seropositivity. The addition of an interaction term between age group and ever hunted wild game to the final model was not significant (*P* = 0.6; OR = 1.1, 95% CI = 0.8–1.4).

## Discussion

The results of this study suggest that ATBF is common in adults in rural areas in Cameroon, and ecological factors associated with the tick vector may increase the risk of *R. africae* infection. The detection of IgG/IgM/IgA and titers as high as 1:2,048 in some participants most likely indicate recent infections with *R. africae* and therefore provide support for the ongoing transmission of infection.

Although the four village sites associated with high odds of *R. africae* seropositivity were widely distributed across Cameroon, three of the four sites were located in lowland rainforest habitats. Lowland rainforest habitats are ideal for *A. variegatum* ticks because of their moderate canopy cover, which provide microclimates favoring tick survival, and high biological diversity, which increase the number of habitats available to the ticks. In addition, lowland rainforest habitats tend to have suitable soils for agriculture and consequently may offer the ticks access to domestic hosts. In contrast, mangrove forest habitats are not preferred by the ticks because they have a low broken canopy cover, relatively lower biological diversity, and brackish or salt water, which provide fewer habitats for the ticks and fewer opportunities for encountering hosts. This study found the expected findings associated with these habitats with most of the lowland forest sites showing high seroprevelances and the mangrove site showing the lowest seroprevalence. However, it is unclear whether these findings are a function of canopy development, biological diversity, presence of domestic hosts, or a combination of these factors.

The highest seroprevalence (51.8%) was recorded in Njikwa, a village in the highland gallery-forest surrounded by cattle-rearing areas. Because Njikwa is not located in the ideal lowland rainforest habitat, this high seroprevalence indicates that the presence of cattle, the preferred host of *A. variegatum*, may be more closely linked with *R. africae* seropositivity than habitat type. In a serosurvey analyzing antibodies reactive to *R. conorii* in Zimbabwe, the highest seroprevalence was recorded in the southern part of the country, where cattle are reared and *A. variegatum* ticks are commonly encountered.^9^ Thus, environmental factors may be less important for *R. africae* seropositivity in areas where domestic hosts are readily available.

It is also possible that an unrecognized tick vector may be transmitting *R. africae* in central Africa. In our previous study, we detected antibodies to spotted fever group rickettsiae in 35% of febrile patients from different localities, particularly urban and suburban communities, where cattle-rearing is rarely practiced and the prevalence of *A. variegatum* is low.^4^ Although the persons examined in that study were a biased group (febrile patients), the results suggest that a vector other than *A. variegatum* could also be transmitting infections in the urban and suburban areas where *A. variegatum* prevalence is low. Recently, *R. rickettsii,* the agent of Rocky Mountain spotted fever, and known to be transmitted by *Dermacentor* species was isolated from the dog tick *Rhipicephalus sanguineus* in Arizona, where febrile patients were diagnosed with Rocky Mountain spotted fever and *Dermatocentor* ticks are uncommon.^22^ Therefire, it could be important to examine other tick species as probable vectors of *R. africae*, especially if the habitats are less hospitable for *A. variegatum.*

In addition to ecological factors associated with seropositivity, this study found that persons 36–45 years of age were more likely to be seropositive than persons of all other age groups. This finding appears to signify the role of certain behavioral or occupational factors pertaining to this age group. Of the three vector-contact-associated activities investigated (wild game hunting, wild pet ownership, and occupations involving agricultural work or direct contact with animals), only game hunting was associated with a moderately increased odds of seropositivity in the multivariable analysis, although it did not reach the level of significance. The interaction between game hunting and age group did not show any significant differences in seropositivity. Thus, game hunting may only partially explain the increased odds of seropositivity in this group of middle-aged adults. The high odds of seropositivity caused by ecological factors as represented by village site may have obscured any smaller significant differences in odds of seropositivity associated with the other vector-contact-associated activities. Nonetheless, these findings suggest that risk factors for ATBF may be similar in indigenous populations in Africa compared with those for international travelers, for whom game hunting has been shown to lead to a 10-fold increased odds of ATBF.^12^

This study had some limitations. Although we analyzed samples for reactivity with *R. africae* only, four rickettsial agents (*R*. *africae*, *R. conorii*, *R. aeschlimannii*, and *R. sibirica mongolotimoniae*) have been detected in Africa. *Rickettsia conorii* is associated with *Rhipicephalus sanguineus* ticks and is mainly encountered in urban areas, and *R. africae* is reported in semi-rural and rural areas.^3^,^8^ Although *R. africae* has not been isolated in Cameroon, we limited this serosurvey to *R. africae* because previous serologic and molecular surveys suggest that *R. africae* rather than *R. conorii* is the rickettsial agent infecting humans in Cameroon.^4^,^5^ In addition, there is evidence that the prevalence of *R. africae* may be as high as 75% in *A. variegatum* ticks in Cameroon.^5^

The strengths of this study include the sample size, which is the largest seroepidemiologic study of *R. africae* infection in native populations in Africa to date, and the evaluation of risk factors for exposure in healthy adults. Previous studies have focused on international travelers and indigenous patients with acute febrile illnesses. Thus, this study extends the findings of these studies to the general population.

Although this serologic evidence further supports previous findings of *R. africae* in Cameroon, isolation of the agent in patients and the vector remains to be achieved. Few studies have analyzed antibodies to *R. africae* by age. Thus, further research is needed to clarify whether high antibody titers are associated with recent infection or the accumulation of antibodies through years of exposure. Active surveillance studies remain important for better determining the magnitude and distribution of African tick bite fever in indigenous populations in Africa.

## Figures and Tables

**Table 1:** Baseline characteristics of the study population (n = 903), Cameroon

Characteristic	No. (%)
Sex		
F	462 (51.2)
M	441 (48.8)
Age group (y)		
16–25	236 (26.1)
26–35	223 (24.7)
36–45	177 (19.6)
46–55	118 (13.1)
> 55	149 (16.5)
Formal education (y)		
0–4	234 (25.9)
4–6	262 (29.0)
7–9	222 (24.6)
> 9	185 (20.5)
House roofing material		
Corrugated tin, tile, or tar with unfinished ceiling	423 (46.8)
Corrugated tin, tile, or tar with finished ceiling	199 (22.0)
Grass or thatched	92 (10.2)
Missing*	189 (20.9)
Village site		
Mundemba	176 (19.5)
Mouanko	151 (16.7)
Lomie	142 (15.7)
Massangan	109 (12.1)
Njikwa	110 (12.2)
Nyabissan	80 (8.9)
Sobia	135 (14.9)
High-risk occupation†		
Neither agriculture nor direct contact	154 (17.0)
Only agriculture	635 (70.3)
Only direct contact	6 (0.7)
Agriculture and direct contact	108 (12.0)
Ever hunted wild game		
No	666 (73.7)
Yes	237 (26.3)
Ever owned a wild pet‡		
No	823 (91.2)
Yes	79 (8.8)
Ever owned as a pet§	
Wild bird		
No	818 (90.6)
Yes	85 (9.4)
Medium-to-large herbivore		
No	728 (80.6)
Yes	175 (19.4)
Pangolin		
No	775 (85.8)
Yes	128 (14.2)
Primate		
No	793 (87.8)
Yes	110 (12.2)
Rodent		
No	695 (77.0)
Yes	208 (23.0)
Reptile		
No	890 (98.6)
Yes	13 (1.4)

* Question not included at the time of questionnaire administration.

† High-risk occupation includes agricultural occupations for subsistence or market, or occupations requiring direct contact with animals, such as butcher, animal breeder, hunter, or veterinary assistant.

‡ Excludes one person with missing data.

§ Medium-to-large herbivore category includes hare, antelope, deer, gazelle, and buffalo; primate category includes monkey, chimpanzee and gorilla; rodent category includes porcupine, hedgehog and rat; reptile category includes crocodile and tortoise.

**Table 2:** *Rickettsia africae* seroprevalence by village site and habitat type, Cameroon

Village site	Habitat type	No. samples	No. (%) *R. africae* positive
Mundemba	Coastal lowland forest	176	26 (14.8)
Mouanko	Mangrove	151	18 (11.9)
Lomie	Congo Basin lowland forest	142	54 (38.0)
Massangan	Highland gallery-forest	109	14 (12.8)
Njikwa	Highland gallery-forest	110	57 (51.8)
Nyabissan	Atlantic equatorial lowland forest	80	23 (28.7)
Sobia	Lowland forest-savanna mosaic	135	51 (37.8)

**Table 3:** Unadjusted and adjusted odds ratios and 95% confidence intervals for risk factors associated with *Rickettsia africae* seropositivity, Cameroon*

Characteristic	No. (%) *R. africae* positive	Unadjusted OR (95% CI)	Adjusted OR (95% CI)†
Sex			
F	134 (29.0)	1.0	1.0
M	109 (24.7)	0.8 (0.6–1.1)	0.9 (0.6–1.4)
Age group (y)			
16–25	48 (20.3)	1.0	1.0
26–35	57 (25.5)	1.3 (0.9–2.1)	4.5 (0.9–2.4)
36–45	63 (35.6)	2.2 (1.4–3.4)‡	1.8 (1.1–2.9)‡
46–55	30 (25.4)	1.3 (0.8–2.2)	1.1 (0.6–1.8)
> 55	45 (30.2)	1.7 (1.1–2.7)‡	1.1 (0.7–1.9)
Formal education (y)			
0–4	75 (32.1)	1.0	
4–6	73 (27.9)	0.8 (0.6–1.2)	
7–9	51 (23.0)	0.6 (0.4–1.0)‡	
> 9	44 (23.8)	0.7 (0.4–1.0)§	
House roofing material			
Corrugated tin, tile, or tar with unfinished ceiling	111 (26.2)	1.0	
Corrugated tin, tile, or tar with finished ceiling	62 (31.2)	1.3 (0.9–1.8)	
Grass or thatched	33 (35.9)	1.6 (1.0–2.5)§	
Missing¶	37 (19.6)	0.7 (0.4–1.0)§	
Village site			
Mundemba	26 (14.8)	1.0	1.0
Mouanko	18 (11.9)	0.8 (0.4–1.5)	0.8 (0.4–1.5)
Lomie	54 (38.0)	3.5 (2.1–6.0)‡	3.1 (1.7–5.6)‡
Massangan	14 (12.8)	0.8 (0.4–1.7)	0.8 (0.4–1.7)
Njikwa	57 (51.8)	6.2 (3.6–10.9)‡	6.3 (3.5–11.5)‡
Nyabissan	23 (28.7)	2.3 (1.2–4.4)‡	2.3 (1.1–4.6)‡
Sobia	51 (37.8)	3.5 (2.0–6.0)‡	3.6 (2.0–6.6)‡
High-risk occupation#			
Neither agriculture nor direct contact	27 (17.5)	1.0	
Only agriculture	175 (27.6)	1.8 (1.1–2.8)‡	
Only direct contact	1 (16.6)	0.9 (0.1–8.4)	
Agriculture and direct contact	40 (37.0)	2.8 (1.6–4.9)‡	
Ever hunted wild game			
No	167 (25.1)	1.0	1.0
Yes	76 (32.1)	1.4 (1.0–2.0)‡	1.4 (0.9–2.2)
Ever owned a wild pet**			
No	217 (26.4)	1.0	
Yes	26 (32.9)	1.4 (0.8–2.2)	
Ever owned as a pet††			
Wild bird			
No	219 (26.8)	1.0	
Yes	24 (28.4)	1.1 (0.6–1.8)	
Medium-to-large herbivore			
No	185 (25.4)	1.0	
Yes	58 (33.2)	1.5 (1.0–2.1)‡	
Pangolin			
No	202 (26.1)	1.0	
Yes	41 (32.0)	1.3 (0.9–2.0)	
Primate			
No	204 (25.7)	1.0	
Yes	39 (35.5)	1.6 (1.0–2.4)‡	
Rodent			
No	177 (25.5)	1.0	
Yes	66 (31.7)	1.4 (1.0–1.9)§	
Reptile			
No	238 (26.8)	1.0	
Yes	5 (38.5)	1.7 (0.6–5.3)	

* OR = odds ratio; CI = confidence interval.

† After likelihood ratio testing and adjustment for confounding, the final model included sex, age group, village site, and ever hunted wild game.

‡ *P* < 0.05.

§ *P* < 0.10.

¶ Question not included at the time of questionnaire administration.

# High-risk occupation includes agricultural occupations for subsistence or market, or occupations requiring direct contact with animals, including butcher, animal breeder, hunter, or veterinary assistant.

** Excludes one person with missing data.

†† Medium-to-large herbivore category includes hare, antelope, deer, gazelle, and buffalo; primate category includes monkey, chimpanzee and gorilla; rodent category includes porcupine, hedgehog and rat; reptile category includes crocodile and tortoise.
